# New Gland Type Discovered in Cestodes: Neurosecretory Neurons Release a Secret into the Fish Host

**DOI:** 10.1134/S0012496623700801

**Published:** 2023-12-19

**Authors:** N. M. Biserova, I. A. Kutyrev, V. V. Malakhov

**Affiliations:** 1 Moscow State University, Moscow, Russia; 2 Institute of General and Experimental Biology, Siberian Branch, Russian Academy of Sciences, Ulan-Ude, Russia

**Keywords:** tapeworms, nervous system, manipulative factors, secretome, glands, exocrine secretion, neurosecretion, parasite–host interactions

## Abstract

Free endings of peripheral neurosecretory neurons (NNs) were found in the tegument of plerocercoids of five species of parasitic cestodes of fish in an ultrastructural study. The free terminals secreted vesicles on the tegument surface and into the host body. Secretion was experimentally shown to increase in response to the host fish blood serum. In the cestode body, NNs form paracrine-type contacts near the cell membranes of the frontal glands, the tegument, and muscles, functioning as endocrine glands. Simultaneously, NNs function as exocrine glands and secrete the so-called manipulative factors, which influence the physiology of the host.

Cestodes (tapeworms) are highly specialized parasites of animals and humans. Nutrients are absorbed through a syncytial outer tissue known as the tegument. A complex mixture of excretory and secretory products, which are collectively termed the secretome, is known to be released by a parasite [1–3]. Terminals of frontal glands penetrate the tegument together with free nerve endings, which are separated from the tegument membrane by a system of specialized contacts. The frontal glands located in the scolex have been described in various cestode orders other than Cyclophyllidea [4–7]. Secretion via an eccrine mechanism has been demonstrated to occur in the cestode frontal glands under the control of brain neurons [8]. Sensory nerve endings that penetrate the tegument are characterized by a rudimentary cilium, a basal body, a root, and other structures found in a dendritic bulb [9]. Several cestode species have free endings that lack traces of a cilium, a kinetosome, and a root, but are separated from the tegumen membrane by a system of specialized contacts [10–13]. The structure and function of these endings remain unknown.

The objective of this work was to study the ultrastructure of free terminals in the tegument in cestode plerocercoids in order to better understand their origin and functional significance.

We examined plerocercoids of five cestode species of the orders Diphyllobothriidea and Bothriocephallidea (Table 1).

**Table 1.** Species examined in the study

No.	Parasite species	Host species	Capture site	Localization in host
1	*Triaenophorus nodulosus* (Pallas, 1781)	*Perca fluviatilis* Linnaeus, 1758	Rybinsk Reservoir	In capsules in the liver
2	*Pyromicocephalus phocarum* (Fabricius, 1780) Monticelli, 1890	*Gadus morhua marisalbi* Derjugin, 1920	White Sea, region of White Sea Biological Station (Moscow State University)	In capsules in the liver
3	*Dibothriocephalus ditremus* (Creplin, 1825) Lühe, 1899	*Oncorhynchus nerka* (Walbaum, 1792)	Lake Kronotskoye, Kamchatka	In capsules on the esophagus, stomach, and pyloric appendages
4	*Dibothriocephalus dendriticus* (Nitzsch, 1824) Lühe, 1899	*Coregonus migratorius* (Georgi, 1775)	Lake Baikal, Kabanskii raion, Buryatia	In capsules on the esophagus, stomach, and pyloric appendages
5	*Ligula alternans* (Rudolphi, 1810)	*Carassius gibelio* (Bloch, 1782) Linnaeus, 1758	Lake Nikitkino, Eravninskii raion, Buryatia	In the abdominal cavity

Ultrastructural studies were performed by transmission electron microscopy, using techniques modified to study cestodes [8, 12, 13]. To assess the changes in activity of secretion from the free terminal surface in *D. dendriticus* and *L. interrupta*, experiments were carried out according to a protocol developed previously [3].

## ULTRASTRUCTURE OF FREE TERMINALS IN THE TEGUMENT

**Fig. 1.**
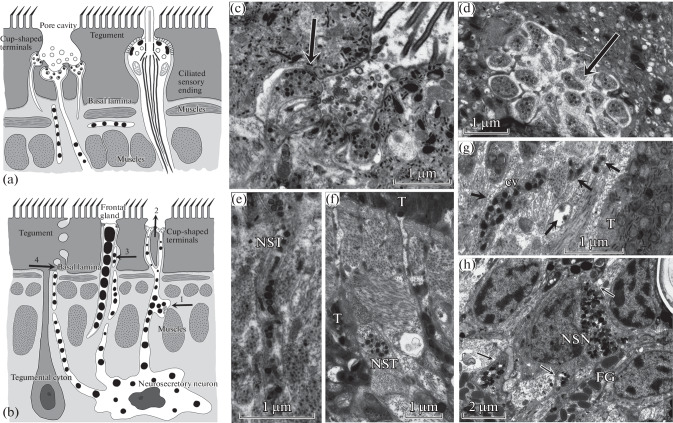
Free terminals in the tegument and neurosecretory neurons (NNs). (a) Structures of ciliary and cup-shaped endings of neurons in the tegument in cestodes. (b) Scheme of exocrine and endocrine secretion by peripheral NNs: 1, neuromuscular paracrine contact; 2, exocrine secretion to the tegument surface; 3, contact with frontal glands of the paracrine type; 4, secretion in the zone of the basal plate of the tegument. (c–e) *Ligula alternans*: (c, d) a group of cup-shaped endings (arrows) with secretory vesicles in the tegument and (e) a longitudinal section of a neurosecretory terminal (NST) included in the tegument. (f) *Pyromicocephalus phocarum*: NST in contact with a tegumental process (T). (g) *Dibothriocephalus dendriticus*: a paracrine contact of NST with muscles under the tegument (arrows); cv, light vesicles. (h) *Pyromicocephalus phocarum*: NN in contact with processes of frontal glands (FG) and muscles (arrows).

Thin tubular free endings with small distal cup-shaped distensions, which contained vesicles, were observed in the tegument in plerocercoids of the five species (Fig. 1). Vesicles are released through the surface membrane into the pore cavity and then to the environment, that is, host tissues. The membrane of a cup-shaped terminal is separated from the tegumental membrane by a narrow septum-containing contact ring, which is underlain by a single indistinct electron-dense support ring on the ending side. Cilia, kinetosomes, and roots were not detected in all of the species examined, and mitochondria were absent from both the cup-shaped terminal and the basal part of the process, which was connected with the perikaryon. In addition, the diameter of the apical distension (450–600 nm) of a cup-shaped terminal was half as large as the diameters of typical sensory endings and terminals of frontal glands in all of the species.

In *T. nodulosus* plerocercoids, an individual pore connected each terminal with the body surface. Pores contained light vesicles of 100–120 nm and single microtubules. The terminal was filled with vesicles, which were released to the tegumental surface, in larvae encapsulated in the perch liver. Free cup-shaped terminals were empty, without vesicles, in plerocercoids isolated from host capsules.

In *P. phocarum* plerocercoids, cup-shaped terminals occurred both individually and in groups. Five to six thin terminals that contained dense vesicles of 90 nm in diameter, branched from the same dendrite, and ended with a common pore were observed in the tegument of a bothrium in *P. phocarum*. It is important to note that vesicles might be present or lacking in the cap of a terminal.

In *D. ditremus* plerocercoid, cup-shaped terminals occurred in pairs and contained light round vesicles of 100 nm in diameter and, sometimes, electron-dense vesicles of 84 nm in diameter. A narrow pore allowed vesicle release on the tegumental surface.

In *D. dendriticus* plerocercoids, two or three cup-shaped terminals contained dense vesicles of 123 nm in diameter and opened into a common pore, which was connected to the environment. The pore structurally differed from the above pores by lacking microtrichia and having a denser structure of the outer tegumental membrane and a greater diameter, which reached 3 µm. Like in the other species, a terminal had a thin support ring on the apical surface and a circular septum-containing contact with the tegumental membrane and contained individual microtubules and vesicles.

In *L. alternans*, cup-shaped terminals were found in the scolex tegument and plerocercoid body. Four terminals opened into a common pore in the tegument and contained short microtubules and dense round vesicles of 90–94 nm in diameter; vesicles were released into the pore cavity (Figs. 1c–1e). In the subtegumental region, wide single dendrites with electron-dense round vesicles branched to produce several free terminals, which opened into a common pore in the distal cytoplasm.

Thus, the cup-shaped terminals are structurally similar in all of the species examined and release secretory vesicles of 100–120 nm into host tissues, acting as exocrine glands. In contrast to sensory endings found in the tegument, the cup-shaped terminals are smaller in diameter; have a single, poorly developed support ring; and release secretory granules on the tegument surface. The cup-shaped terminals ultrastructurally differ from frontal gland ducts by lacking peripheral microtubules and having small vesicles as their secretory material.

## ULTRASTRUCTURE OF NEUROSECRETORY NEURONS

Peripheral NNs were found in the subtegumental region and cortical parenchyma in all of the species examined (Figs. 1b, 1f–1h). NN bodies were elongated irregular in shape and had several neurites, which were filled with round electron-dense neurosecretory vesicles or granules (NSGs). The NSG diameter varied from 90 to 140 nm. NN processes lacked mitochondria and peripheral microtubules in contrast to frontal gland ducts. The cytoplasm was dense, looked granulated, and contained ribosomes neurosecretory processes (NSPs) in contrast to dendrites and axons of CNS neurons. In *D. dendriticus*, the NSP density is 9–10 per 500 µm^2^ in the subtegumental region.

NN processes often went along myofibrils of circular and longitudinal muscles of the coating and were sometimes tight against them. Small varicosities formed in the contact regions and contained not only NSGs, but also groups of round light vesicles. Secretory material was released into the intercellular space near the myofibril via a paracrine mechanism. A presynaptic thickening and a T-shaped membrane fold, which marks a synaptic contact, were absent.

We observed for the first time that NN processes formed contacts with processes of frontal gland cells that were strengthened by peripheral microtubules in all of the species examined. The NSG diameter was 140–160 nm on average in the contact region. In *D. ditremus* plerocercoids, NSP contacts with frontal gland terminals were detected directly in the terminal pore of a secretory duct in the tegument (Fig. 1b). The NSG diameter varied from 90 to 130 nm in the contact region. Thus, NN processes are capable of penetrating the distal cytoplasm of the tegument along with frontal gland ducts and releasing neuroactive substances into host tissues. In *P. phocarum*, *D. dendriticus*, and *L. alternans*, we observed the NSPs that penetrated the tegument, contained electron-dense vesicles (90 nm), and released their secretory material into the pore cavity. Cup-like terminals found in the tegument were NN processes in these cestodes. Thus, part of peripheral NNs release their secretory material to the outside, into the host body, acting as exocrine glands.

## EXPERIMENTAL STUDY OF SECRETORY ACTIVITY WITH HOST BLOOD SERUM

Tapeworms were incubate for 3, 6, 12, and 24 h. In *D. dendriticus*, cup-shaped terminals were depleted of vesicles, the terminal diameter decreased, and the granule diameter increased in NNs after 3-h incubation. Secretion increased and the number of terminals per pore increased to three or four after 6-h incubation. Signs of exhaustion and cup narrowing were observed in terminals after 12-h incubation, and deformities of terminals in the tegument were detected after 24-h incubation. Thus, neurosecretion was maximum after 6-h incubation and was exhausted by 24 h of incubation in *D. dendriticus*.

In *L. alternans*, 3-h incubation led to more intense secretion from the surfaces of cup-shaped terminals and increased the number of dense vesicles per terminal to 20 and the number of NSPs in the regions of the basal plate and muscles of the tegument, where secretory material was released via a paracrine mechanism. Intense vesicle secretion continued after 6-h incubation, and the number (four or five) and diameter of terminals in a pore were increased. Numerous NSPs were observed near the basal plate and muscles of the tegument; the basement membrane of the tegument formed many vacuoles, which were filled with a fibrillary matrix, moved to the apical membrane, and released their contents to the outside. After 24-h incubation, the terminal diameter decreased, but NSPs grew in number and contained more granules in the subtegumental region; some of the processes were directed toward the basement membrane. Thus, secretion and the NSP number in *L. alternans* increased starting from 3 h of incubation and adaptation to loading occurred by 24 h.

Free terminals without signs of ciliary and root systems have been described in several cestode species [6, 10–13]. A concave shape of the apical cup with a single support ring is characteristic of the free terminals in all of the species examined. According to our findings, the cup diameter is at least twice smaller than that in ciliary and cilium-free sensory terminals. The question arises as to whether the cup-shaped free terminals serve as sensory dendrites or are involved in neurosecretion of manipulative molecules into host tissues. Two NN populations have been observed in the scolex in various cestode species, one being associated with CNS and the other, with PNS [8, 14–18]. Our study showed that, in the five cestode species examined, PNS-associated NNs have processes that release secretory material via a paracrine mechanism into the intercellular space near muscle cell membranes, frontal gland cells, and the tegument and on the tegumental surface into host tissues. The presence of endocrine glands in cestodes is a matter of discussion [15, 16]. Our findings indicate that NNs of the cestode PNS may act both as endocrine glands with secretion into the intercellular space and as exocrine glands with secretion onto the tegumental surface. The fact that secretion increases in response to the host blood serum suggests an important exosecretory function of NNs, which represent a new gland type in cestodes. Neurosecretory material released from free terminals of cestodes into the fish body may act as a manipulative factor that affects the endocrine system in the vertebrate host.
